# Development and Validation of a Brief Digital Cognitive Screening Tool for HIV-associated Neurocognitive Disorder in Lima, Peru

**DOI:** 10.21203/rs.3.rs-11166435/v1

**Published:** 2026-09-28

**Authors:** Monica M. Diaz, Marleny Nolasco, Kimberly Enders, Rebecca K. Jenkins, Valeria Roldan, Sofia Tovar-Ramirez, Yamile Rodriguez-Angeles, Fernando Mejia, Katie Mollan, Gwenn A. Garden, Maria J. Marquine, Patricia J. Garcia, Victor Valcour, Katherine L. Possin, Elena Tsoy

**Affiliations:** University of North Carolina at Chapel Hill School of Medicine; University of North Carolina at Chapel Hill; University of North Carolina at Chapel Hill; University of North Carolina at Chapel Hill; Cayetano Heredia University; Cayetano Heredia University; Cayetano Heredia University; Cayetano Heredia University; University of North Carolina at Chapel Hill; University of North Carolina at Chapel Hill; Duke University; Cayetano Heredia University; University of California, San Francisco; University of California, San Francisco; University of California, San Francisco

**Keywords:** digital cognitive screening tool, HIV associated neurocognitive disorder, Peru, Latin America, Spanish

## Abstract

**Background:**

HIV-associated neurocognitive disorder (HAND) remains a frequent yet under-recognized complication among people with HIV (PWH), even while on antiretroviral therapy. In Peru, feasible and accurate brief cognitive tools are needed for underserved clinical settings.

**Methods:**

We recruited Spanish-speaking adults with and without HIV in Lima, Peru. Eleven TabCAT tests were evaluated in a development sample, with demographic norms derived from adults without HIV. A four-test battery with an estimated administration time of 14 minutes was evaluated against a comprehensive paper-and-pencil reference battery in an additional sample of PWH. Concurrent validity was examined using correlations between cognitive tests in the same domain.

**Results:**

Participants included 293 PWH (29.0% female; age [mean ± SD] 47.2 ± 11.8 years; education 12.8 ± 2.7 years) and 162 HIV-negative healthy adults (53.1% female; age 46.5 ± 14.2 years; education 13.6 ± 2.7 years). The four-test battery comprised Birdwatch, Match, Set Shifting, and Rapid Naming. Its area under the receiver operating characteristic curve (AUC) was 0.797 (95% CI: 0.691, 0.903; n = 115) in development and 0.819 (95% CI: 0.740, 0.899; n = 150) in validation samples. Validation sensitivity was 81.6% and specificity was 70.5%. Digital tests exhibited domain-specific moderate correlations with reference paper-and-pencil tests demonstrating concurrent validity.

**Conclusions:**

This brief battery shows promise for cognitive screening among literate, Spanish-speaking PWH in urban Peru. However, it does not establish a clinical HAND diagnosis. Further evaluation of interpretation thresholds, lower literacy levels and rural populations, and referral pathways is needed before routine implementation of this tool.

## Introduction

HIV-associated neurocognitive disorder (HAND) is a frequently underrecognized condition in which people living with HIV (PWH) develop cognitive impairment with significant clinical, social, and economic implications(1,2). Despite improved life expectancy with widespread access to antiretroviral therapy (ART), cognitive impairment continues to affect PWH, including those with effective viral suppression(3). Risk factors for HAND include older age, low socioeconomic status, a low CD4 + T-cell nadir, poor ART adherence, and comorbid conditions, such as hypertension, depression, HIV-associated coinfections, and substance use(4,5,6,7), all of which contribute to increased morbidity, mortality, and healthcare burden globally(8,9). With the advent of ART, however, the clinical phenotype of HAND has shifted to include deficits predominantly in executive function, attention, and processing speed(10,11). This overlap between cortical and subcortical profiles complicates the selection of appropriate neuropsychological screening tools for HAND that can provide both sensitivity to early detection and scalability with regard to time available in the clinic and resource demands. The International HIV Dementia Scale (IHDS), for example, showed poor discrimination of cognitive impairment in a study in East Africa(12).

In Peru, over 90,000 individuals currently live with HIV(13). While 76% of them receive treatment and care through the National HIV/Sexually Transmitted Infection (STI) Program of the Peruvian Ministry of Health(14), there are currently no national guidelines addressing the screening or management of non-transmissible complications of HIV, such as HAND. Thus, integrating appropriate screening tools for HAND into the national HIV care program in Peru could help identify patients who need further cognitive evaluation and support appropriate treatment and risk factor management.

Assessment for HAND relies on a comprehensive clinical evaluation informed by paper-and-pencil neuropsychological testing(15). A comprehensive battery can require several hours, and limited access to trained neuropsychologists and psychometrists constrains its use in routine care in Peru(16). Neuropsychological batteries have been evaluated in Spanish-speaking PWH, including adults with limited formal education(17). However, evidence from one Spanish-speaking population does not establish applicability to all Peruvian settings. These challenges motivate evaluation of a brief screening battery that can identify patients who may benefit from more comprehensive assessment(18,19).

Digital neuropsychological platforms, such as the Tablet-based Cognitive Assessment Tool (TabCAT), offer unique opportunities to administer cognitive testing in primary care settings as they do not require administration by highly trained specialists yet maintain standardization and precision of measurement. The TabCAT platform includes tests measuring distinct cognitive domains, many of which have been designed or adapted for cross-cultural applicability in Latin American populations(20). TabCAT tests showed excellent sensitivity and specificity in detecting mild cognitive impairment and dementia in English and Spanish speaking older adults without HIV in the U.S.(21,22), Cuba(23), and Peru(24) but have not yet been validated to detect HAND in PWH. In this study, we sought to develop and validate a brief neuropsychological battery of TabCAT tests for cognitive screening among PWH in Lima, the capital city of Peru.

## Methods

### Participants

We recruited PWH and people without HIV (PWoH), who were at least 18 years of age, native Spanish speakers, had Peruvian nationality, and completed at least primary school (5–6 years of formal education). PWH were recruited from a public hospital clinic (Hospital Cayetano Heredia) of the Ministry of Health that attends PWH in Lima, Peru and from a local non-governmental organization that aids PWH based in Lima. These participants had confirmed HIV infection using OnSite HIV 1/2 Antibody Plus Combo rapid antibody testing, a lateral flow immunoassay for detection of HIV-1 and 2 antibodies (IgG, IgM and IgA), were continuously on ART for ≥ 1 year, and had a plasma HIV RNA < 40 copies/ml within the past 6 months. PWoH were recruited through flyers posted in the study hospital or as referrals from family members or friends of PWH who were enrolled in the study. PWoH had an HIV-negative test on the study visit date or within two weeks prior to the study visit.

Participants were excluded for self-report of neurological conditions that could account for cognitive impairment, including sequelae of stroke, uncontrolled epilepsy, traumatic brain injury, or central nervous system opportunistic infection; self-reported untreated psychiatric illness; self-reported substance use disorder involving alcohol, marijuana, or other illicit substances, assessed with reference to DSM-5 criteria(25); or illiteracy (assessed by asking if the individual was able to read and write). The study neurologist (MMD) reviewed cases in which eligibility was uncertain.

### Procedures

All participants completed paper-and-pencil and TabCAT neuropsychological assessments in a private clinic room, administered by examiners (STR, YRA, VR) trained by the study neurologist (MMD). A native Spanish-speaking, U.S.-licensed neuropsychologist (MJM) provided training and ongoing supervision for paper-and-pencil test administration and scoring. Quality control reviews were conducted every other month by the principal investigator (MMD) for both modalities, with any issues addressed collaboratively through consensus with the study neuropsychologists (ET, MMD). Participants also completed standardized demographic questionnaires that collected information on age, sex assigned at birth, and years of education. All participants received their formal education within the Peruvian national schooling system.

### Candidate TabCAT Tests

TabCAT is a software platform developed at the University of California, San Francisco (UCSF), for administering, interpreting, and scoring cognitive and other clinical assessments. We evaluated 11 candidate tests using Spanish versions that had been translated and adapted before this study. The adaptation committee included two Peruvian experts. Translation and cultural adaptation are distinct from validation in a specific population; the present study evaluated test performance and derived local demographic norms. Adaptation procedures followed the general guidance by the International Test Commission(26) and neuropsychology-specific recommendations by Nguyen and colleagues(27). The candidate tests were:

Birdwatch is a 7-minute test of visual associative learning and memory. Participants are shown pairs of visual stimuli (birds and landscapes) and are asked to remember individual stimuli with their correct pair. The task uses a selective reminding paradigm and an adaptive design. The primary measure is the total number of correct responses across seven trials. Birdwatch was introduced after the first 39 participants had enrolled given Birdwatch became available partway through the study’s enrollment. Those participants who had not received it at their baseline visit received it at the time of their one-year follow-up visit together with the other digital tests. Birdwatch data from each participant was only utilized from the first administration whether at baseline or follow-up visit. For all other tests described below, only the baseline test administration data was used.

Favorites is a 5-minute test of multimodal associative memory. Participants are shown pairs of verbal (words) and visual (faces) stimuli and are later asked to recall these pairs. The primary measure is the total number of correct responses across two immediate recall trials and one 10-minute delayed recall trial.

Dot Counting is a 7-minute test of verbal working memory, during which participants are shown an array of geometric shapes and are asked to count and remember the number of shapes of a specific type. The primary measure is the total number of correct responses across six trials.

Flanker is a 4-minute test of cognitive control and inhibition. Participants are shown a row of five arrows and are asked to quickly indicate the direction of the arrow in the center surrounded by congruent or incongruent arrows. The primary measure is the score combining accuracy and reaction time performances on incongruent trials only.

Match is a 2-minute test of processing speed, attention, and working memory modeled after the classic digit symbol matching paradigm. Participants are asked to quickly match simple abstract pictures with single digit numbers based on a fixed key. The primary measure is the total number of correct responses within 2 minutes.

Set Shifting is a 4-minute test of executive task switching, during which participants are asked to quickly match a target stimulus to one of two similar stimuli based on set instructions. The primary measure is a score combining accuracy and reaction time results on shifted trials only.

Tempo is a 3-minute test of rhythm keeping. Participants are presented with a metronome-like auditory tone and are asked to tap the screen at the same rate. The primary measure is the degree of discrepancy between a participant’s tapping rate and the target tone across five trials.

Line Length and Line Orientation are 2-minute adaptive tests of visuospatial discrimination. Participants are asked to identify a longer line out of two displayed (Line Length) or a line parallel to the target line out of two displayed (Line Orientation). The primary measures are scores representing the average length (Line Length) or angle (Line Orientation) difference between target lines, at which probability of correct responses is 75%.

Rapid Naming is a 1-minute test of speeded confrontation naming. Participants are shown line drawings of everyday objects and are asked to name each object as quickly as they can. The primary measure is the total number of correct responses in one minute.

Quick Tap is a 2-minute task of fine motor speed and dexterity. Participants are asked to tap the index finger of their non-dominant hand repeatedly as fast as they can. The primary measure is the total number of taps across five trials.

TabCAT scores were not used to assign the reference HAND classification. Classification was based on the comprehensive paper-and-pencil battery and the reference diagnostic criteria described below assigned by the biostatisticians (RJ, KE, KM) based on the established Antinori 2007 HAND criteria(1).

### Norming of Candidate TabCAT Tests

Regression-based norms were derived separately for each primary TabCAT measure using available complete cases among the 162 PWoH in the development sample; test-specific sample sizes are reported in Supplementary Table 1. Candidate demographic predictors were age, years of education, and sex assigned at birth. Quadratic age and education terms were examined but were not included in the reported models. Backward elimination stopped when all remaining predictors had p < 0.05 or when two predictors remained. The final predictor sets varied across tests. Two-way interactions among retained predictors were examined but omitted after considering their statistical evidence and incremental explained variance.

For each test, predicted scores $\left(\hat{y}\right)$ were obtained from the final regression model. Standardized residuals were calculated as $z=\frac{y-\hat{y}}{RSE}$, where $RSE=\sqrt{\frac{\sum {\left(y-\hat{y}\right)}^{2}}{n-p}}$. Regression-based normative models for the TabCAT measures are presented in Supplementary Table 1. Here, n is the test-specific normative sample size and p is the number of estimated regression parameters, including the intercept.

#### Reference Neuropsychological Tests

We selected a comprehensive neuropsychological battery assessing seven domains: attention/working memory, executive function, learning and memory, fine motor skills, processing speed, language, and visuospatial skills. Test selection considered psychometric evidence and alignment with the 2007 HAND research criteria(15), including measures used in the AIDS Clinical Trials Group battery(28). Relevant Spanish-language normative and validation work includes NP-NUMBRS(17,29), Latino norms for the Benson figure within the Uniform Data Set battery(30), and Spanish Color Trails norms(31). These studies support assessment in their respective populations; the scores used here were adjusted using previously developed Peruvian reference-battery norms reported in a preprint(32).

The primary scores from the reference neuropsychological battery included: Color Trails 2 Total Time(31) (executive function); Wechsler Adult Intelligence Scale-III (WAIS-III) Digit Span(33) Forward and Backward Total Score (attention/working memory); Grooved Pegboard Total Time(34) (fine motor skills); Hopkins Verbal Learning Test – Revised(35,36) (HVLT-R) Sum of Learning Trials and Delayed Recall Scores, Benson Complex Figure Delayed Recall Total Score(30) (learning and memory); Benson Complex Figure Copy Total Score (visuospatial); Color Trails 1 Total Time(31), WAIS-III Digit Symbol Coding Total Correct(33), and WAIS-III Symbol Search Total Correct (processing speed); and Letter Fluency (“PRM”) and Animal Fluency Total Correct (language).

### Diagnosis of HAND

Demographically adjusted neuropsychological test results from the comprehensive paper-and-pencil battery were used to classify participants as meeting or not meeting HAND criteria based on 2007 HAND nosology(15). Under this framework, HAND requires performance at least 1 SD below the demographically adjusted mean in two or more cognitive domains, after excluding alternative causes of neurocognitive dysfunction. Participants without HIV who had a mean z-score < −1 in two or more domains were diagnosed with cognitive impairment.

### Statistical Analyses

#### Development of a 15-minute TabCAT Tool

The development sample included 142 PWH and 162 PWoH. Candidate TabCAT scores were standardized using the local regression-based norms described above (Supplementary Table 1). Eleven separate single-predictor analyses evaluated discrimination of the reference HAND classification, one for each candidate measure. Available cases were used for each test. The four selected measures were evaluated together in complete-case multivariable analyses. Youden’s index was used to select the optimal classification threshold, which represents the z-score cutoff in the single test analyses and the predicted probability cutoff in the multivariate analysis. Confidence intervals were calculated using DeLong’s method for AUC and the exact Clopper-Pearson method for sensitivity and specificity.

Logistic regression estimates for the four-test battery are reported as adjusted odds ratios in Supplementary Tables 2 and 3.

The intended battery duration was no more than 15 minutes. Initial test selection considered discrimination, cognitive domain coverage, domain overlap among measures, and administration time. Within each domain, tests with the highest AUC were prioritized for selection in the 15-minute brief battery; in particular, the executive function domain was prioritized for inclusion given its clinical relevance to HAND. Though Dot Counting had a slightly higher AUC, Set Shifting was selected due to shorter administration time and to ensure executive functioning was represented in the final model. Two additional tests with higher AUC than Set Shifting, Flanker and Favorites, were not included in the final model due to domain overlap with tests with higher AUCs. Estimated test durations are summarized in Supplementary Table 5.

#### Validation of the 15-minute TabCAT Tool

The four selected tests (Birdwatch, Match, Rapid Naming, and Set Shifting) were administered in an additional sample of 151 PWH. Concurrent validity was examined using Pearson correlations between TabCAT and reference tests assessing related domains, pooling the development and validation samples. Spearman rank correlations were also examined to assess sensitivity to extreme observations (Supplementary Fig. 1 and Supplementary Table 4). Criterion-related discrimination was evaluated in the additional sample using the four TabCAT measures as predictors and the reference HAND classification as the outcome, with logistic regression and discriminant analyses and ROC curves. The target AUC was 0.80 or greater. Multivariable analyses used participants with all four test scores available. For the four-test battery, the ROC operating points shown in Fig. 2 were selected using Youden’s index (sensitivity + specificity − 1).

## Results

### Participant Characteristics

The study included 455 participants: 162 PWoH and 142 PWH in development, and 151 PWH in validation (Table 1). HAND criteria were met by 43/142 PWH (30.3%) in development and 38/151 (25.2%) in validation. Mean age was 46.5, 46.2, and 48.2 years in the three groups, respectively; mean education was 13.6, 12.8, and 12.7 years. The proportion assigned female sex at birth was 53.1% among PWoH, 31.7% among development PWH, and 26.5% among validation PWH. Normative models used 155–162 PWoH per test based on completion (Supplementary Table 1). Age was retained in 10 models, education in 9, and sex in 4; the selection procedure retained at least two predictors, including some whose 95% confidence intervals included zero.

### Development of a 15-minute TabCAT Tool

Rapid Naming (AUC = 0.731) and Match (AUC = 0.727) had the largest individual AUCs in the corrected development analyses (Table 2). The selected brief battery comprised Birdwatch (associative learning and memory), Match (processing speed, attention, and working memory), Set Shifting (executive task switching), and Rapid Naming (speeded confrontation naming). The estimated combined test duration was 14 minutes, excluding transitions and additional instruction time (Supplementary Table 5). Dot Counting, Favorites, and Flanker had higher individual AUCs than Set Shifting in the corrected analyses but were not selected for the battery due to domain overlap or greater than desired time demands. The four-test development analysis included 115 PWH and yielded an AUC of 0.797 (95% CI: 0.691, 0.903), sensitivity of 0.700 (95% CI: 0.506, 0.853), and specificity of 0.894 (95% CI: 0.808, 0.950) (Fig. 2).

### Concurrent Validity of the Tests in the 15-minute TabCAT Tool

Birdwatch correlated with Benson Complex Figure Delayed Recall (r = 0.43, n = 429) and HVLT-R Delayed Recall (r = 0.51, n = 428). Set Shifting correlated with Color Trails 1 (r = −0.41, n = 447) and Color Trails 2 (r = −0.45, n = 447). Match correlated with Color Trails 1 (r = −0.59, n = 454) and Color Trails 2 (r = −0.61, n = 454). Rapid Naming correlated with Animal Fluency (r = 0.50, n = 455) and Letter Fluency (r = 0.48, n = 455). All Pearson p-values were < 0.001. Spearman correlations were similar in direction and broadly similar in magnitude, supporting the same overall pattern (Supplementary Fig. 1 and Supplementary Table 4).

### Criterion Validity of the 15-minute TabCAT Tool

The complete-case four-test analysis in the validation sample included 150 PWH (Table 3). The AUC was 0.819 (95% CI: 0.740, 0.899), sensitivity was 0.816 (95% CI: 0.657, 0.923), and specificity was 0.705 (95% CI: 0.612, 0.788) at the reported operating point (Fig. 2). These estimates describe discrimination relative to the reference HAND classification. Table 3 summarizes ROC performance; Supplementary Tables 2 and 3 report adjusted odds ratios for the four-test models.

## Discussion

In this study of 293 PWH and 162 PWoH in Lima, the brief TabCAT battery showed concurrent validity with related paper-and-pencil tests and an AUC of 0.819 in the additional PWH sample. Sensitivity of 81.6% and specificity of 70.5% suggest potential screening utility, with both false-negative and false-positive results expected. The study also provides local regression-based norms for the candidate measures, derived from 162 PWoH. The demographic associations reported in Supplementary Table 1 support consideration of age and education when interpreting scores. Our study provides necessary evidence for validation of this tool in local settings, highlighting its potential to perform well in a Latin American HIV clinic context as a scalable approach to early HAND detection in this region. Our approach additionally delivers a rigorous and systematic framework for the development of TabCAT norms for Spanish speakers, with particular relevance to those in Latin America. Across the 11 TabCAT tests, age was retained in 10 normative models, education in 9, and sex in 4. This methodology provides a robust way to generate locally-derived norms for application in young and middle-aged urban-dwelling adults in Latin America.

The present study builds on established approaches to cross-cultural cognitive assessment rather than introducing a new adaptation methodology. Prior studies have used community-informed adaptation of TabCAT measures in Nigeria, linguistic adaptation and reference battery validation of NeuroScreen among South African adults with HIV, and local regression-based standardization of TabCAT-BHA in older Peruvian adults(24,37,38). This study contributes an evaluation of a brief combination of TabCAT measures for cognitive screening among Spanish-speaking adults with HIV in urban Peru. Using previously adapted Spanish language tests, we evaluated 11 candidate measures, derived local demographic norms from adults without HIV, and assessed the four-test battery against a comprehensive paper-and-pencil reference battery in an additional sample of adults with HIV. This approach provides evidence about performance in the intended clinical population rather than assuming that findings from other populations or diagnostic contexts are transferable to this particular population. Consequently, our findings support the potential utility of this battery in the studied population but do not establish methodological superiority or readiness for routine clinical implementation which should be evaluated in a future study.

The composition of the final battery is consistent with the multidomain cognitive phenotype of HAND, with particularly strong representation of processing speed and executive functioning. Match and Rapid Naming showed the greatest individual discrimination of HAND in the development sample, while Match and Set Shifting showed the strongest discrimination in the independent validation sample. Notably, Match and Set Shifting assess speeded executive processes, domains that are commonly affected among people with HIV in the ART era (11). Birdwatch, an associative learning and memory task, also demonstrated consistent discrimination across samples, supporting the contribution of learning and memory to detection of HAND. Rapid Naming showed promising discrimination in the development sample, although its individual performance was more modest in the validation cohort. As a timed lexical retrieval task, Rapid Naming may reflect not only language retrieval but also the efficiency and speed of accessing well-learned information (39), although further work is needed to understand which cognitive processes account for its association with HAND. Therefore, the selected measures suggest that brief assessment across complementary cognitive domains, rather than reliance on a single cognitive construct, may be useful for identifying neurocognitive impairment among people with HIV.

Cognitive screening instruments developed in North America or Europe may not translate well into low- and middle-income country (LMIC) settings(40,41). For example, one systematic review found that many commonly used cognitive screening tests, such as the Mini-Mental State Examination, are strongly influenced by years of schooling and literacy level, limiting validity in populations with heterogeneous educational backgrounds(42). Moreover, many tools do not account for differences in demographic characteristics, educational attainment, literacy, language, and cultural background, which may lead to inaccurate interpretation of test results(43,44).

Strengths of this study include the use of previously adapted Spanish language tests, a local normative sample of 162 PWoH, and evaluation in an additional sample of PWH. Participants had completed at least primary school, and mean education among the normative participants was 13.6 years. Prior Peruvian research evaluated TabCAT in older adults(24), whereas the present study examines its use for screening PWH age 18 years of age or older. However, acceptability, accessibility, implementation feasibility, and the comparative accuracy of different normative approaches were not directly assessed in this study. Our overall Peruvian sample of 455 had a mean educational level of 13.1 years, but educational levels ranged as low as 6 years of education. We therefore selected tests that are less reliant on high literacy or Western-culture specific stimuli from the TabCAT platform. Our results supporting linguistic and cultural acceptability and accessibility of TabCAT tests complement prior work in Peru that validated several TabCAT tests for older adults in rural and urban settings (24).

In many LMIC settings, cognitive testing using a tablet introduces challenges, including differing levels of familiarity with touchscreen and digital platforms and culturally specific concepts embedded in translated instruments that can underestimate the validity of a cognitive screening instrument (45,46,47,48). Locally derived norms may help contextualize performance, but demographic adjustment does not eliminate these influences or establish the cause of a low score. This highlights the need to utilize locally derived norms from local healthy populations rather than apply norms derived from other populations that may lead to over-estimation of cognitive impairment, as demonstrated in South Africa where U.S. norms yielded a 62.2% impairment rate among neurologically intact adults compared with 15.0% using South African norms (49). This study used previously adapted Spanish TabCAT versions and locally derived norms, but did not undertake a new Spanish translation or directly compare local and foreign norms. The benefit of local norms relative to other reference populations requires direct evaluation in other studies(49).

The findings support further evaluation of a brief digital screening approach in Spanish-speaking PWH. Clinical diagnosis still requires assessment of functional status, medical and psychiatric comorbidity, substance use, and other potential causes of cognitive difficulties with an appropriately interpreted neuropsychological evaluation(4,15). A positive screen should prompt clinical assessment rather than be treated as a definitive HAND diagnosis. A negative screen should not preclude assessment when cognitive symptoms or functional decline are present or reported. Although short administration and automated scoring may help with clinic workflow, feasibility in the participating clinics still needs to be measured in future studies.

Implementation within the primary care HIV clinic setting would require an assessment of existing specialist referral pathways and investment in access to neuropsychologists and trained examiners, as well as evaluation of staffing, digital familiarity, and follow-up capacity. Prior HIV clinic studies support the feasibility of tablet screening in their own settings(50,51), but this remains to be established locally. Predictive values also depend on prevalence of HAND: using the reported validation sensitivity and specificity, an illustrative HAND prevalence of 25.2% gives a positive predictive value of approximately 48% and a negative predictive value of 92%. At an assumed prevalence of 10%, these are approximately 24% and 97%. These calculations assume unchanged sensitivity and specificity and are not observed results from a primary care implementation study.

Several limitations affect interpretation of our results. The sample was recruited in urban Lima and excluded people who were illiterate or had not completed primary school; the findings should not be generalized to those groups, including patients attending the same clinics, or to rural populations without further study. The cross-sectional design does not establish longitudinal predictive validity or clinical benefit. The four-test development analysis included 115/142 PWH, leaving 27 (19.0%) outside that analysis. Birdwatch was introduced after the first 39 participants had enrolled, contributing to missingness, and some early participants received it at a later visit. The resulting timing differences and incomplete data may bias estimates. Initial normative sample and HAND domain errors were corrected after the brief battery had been selected, and unavailable validation tests prevented reassessment of alternative batteries. Finally, data-dependent demographic selection, including the requirement to retain at least two predictors, may produce sample-dependent norms. Sensitivity analyses retaining age, education, and sex in every model and evaluation of a prespecified scoring rule and cutoff in a new sample are needed in future studies.

In conclusion, the four-test TabCAT battery shows promise for cognitive screening among literate, Spanish-speaking PWH in urban Peru. Its role is to identify patients who may need further comprehensive clinical assessment of HAND. Integrating such tools into HIV programs offers a unique opportunity to screen for HAND early-on and intervene by risk factor reduction and treatment. Future work should extend validation to rural and more diverse education and literacy contexts, determine longitudinal predictive value of the tool, and test implementation strategies into the HIV clinic workflow in Peru to embed screening and referral pathways into HIV care systems.

## Supplementary Material

Supplementary Files

This is a list of supplementary files associated with this preprint. Click to download.

- SupplementaryTablesPeruTabcat09.14.2026.docx

## Figures and Tables

**Figure 1:**
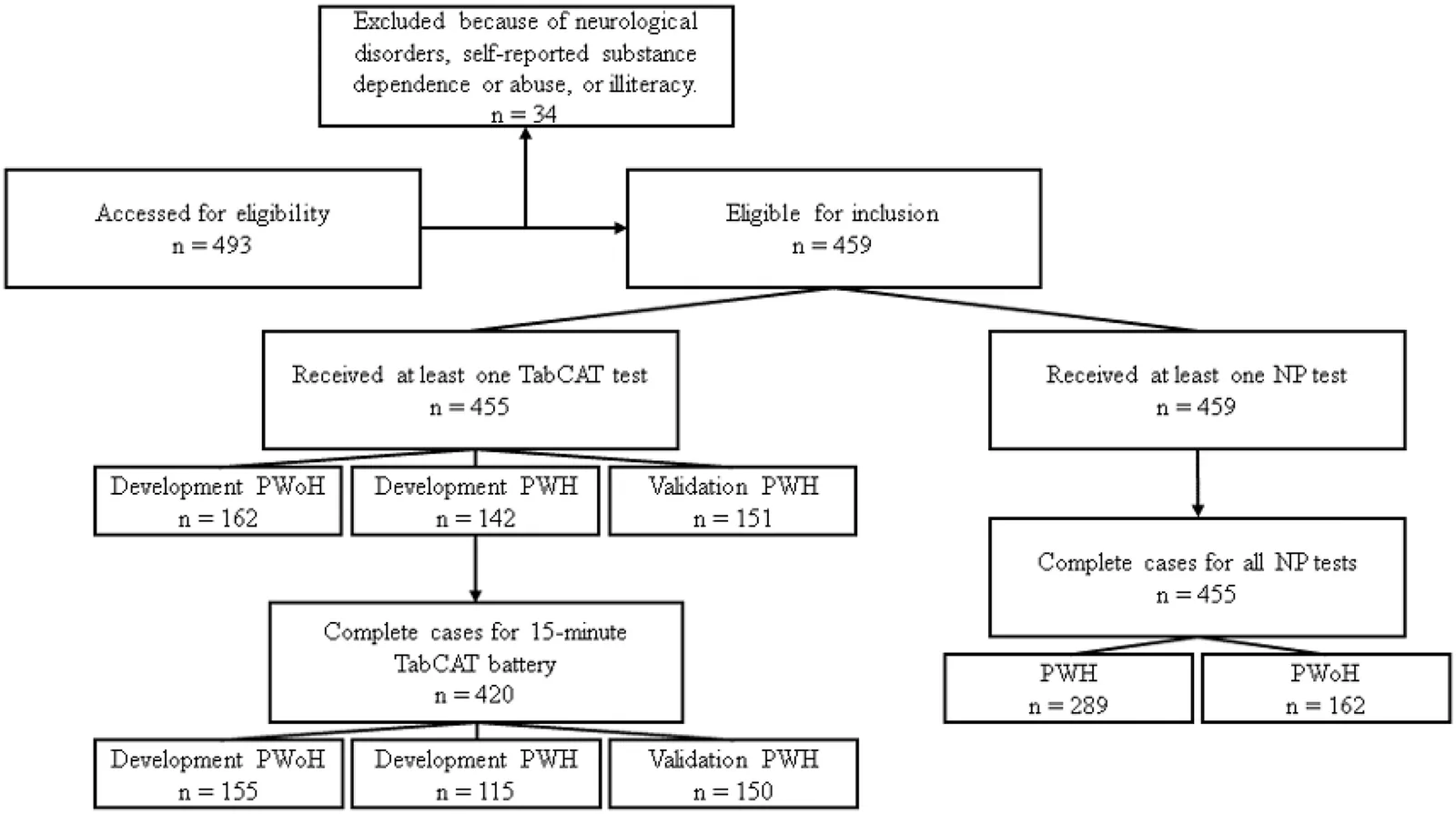
Participant flow diagram; NP refers to the reference neuropsychological tests.

**Figure 2:**
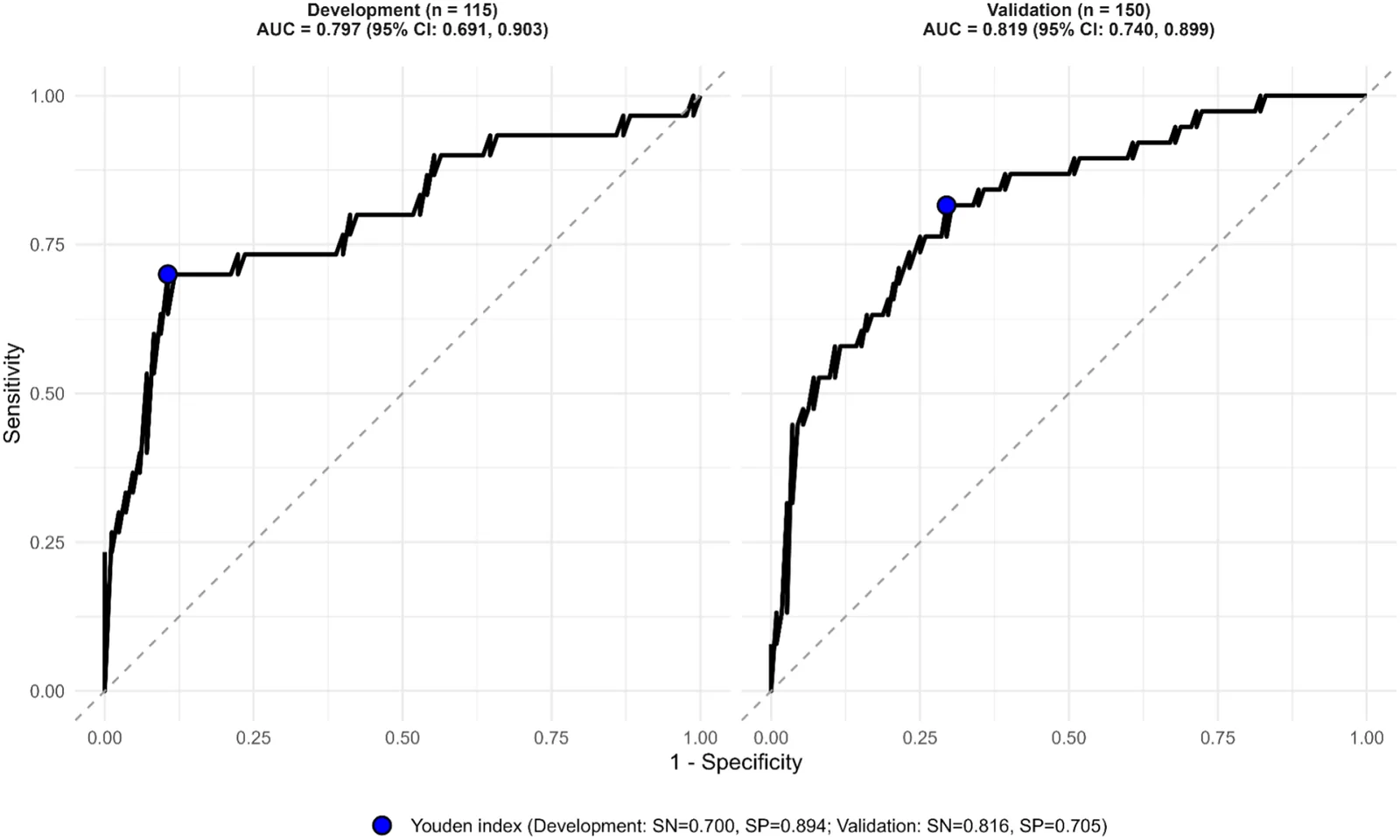
Receiver operating characteristic curves predicting HAND using a 15-minute TabCAT test battery in PWH

**Table 1:** Demographic characteristics and reference neuropsychological test scores by HIV status in the development and validation samples (n = 455)

Variable Mean ±SD or n (%)	Development Sample		Validation Sample
	PWoH (n = 162)	PWH (n = 142)	PWH (n = 151)
Age (years)	46.5 (14.2)	46.2 (13.0)	48.2 (10.4)
Female Sex at Birth	86 (53.1%)	45 (31.7%)	40 (26.5%)
Education (years)	13.6 (2.7)	12.8 (2.9)	12.7 (2.6)
Diagnosis of HAND	N/A	43 (30.3%)	38 (25.2%)
Animal Fluency (Total correct)	22.3 (5.3)	21.1 (5.8)	21.5 (5.5)
Benson Complex Figure Copy	15.5 (1.0)	15.3 (1.3)	14.9 (1.4)
Benson Complex Figure Delayed Recall	12.0 (2.7)	11.7 (2.5)	11.3 (3.1)
Color Trails 1 (Total time)	52.9 (23.6)	58.8 (27.8)	55.3 (24.1)
Color Trails 2 (Total time)	101.4 (44.7)	117.7 (69.1)	104.4 (39.2)
Letter Fluency “PMR” (Total correct)	35.1 (10.5)	34.8 (11.5)	36.5 (13.7)
Grooved Pegboard – Dominant (Total time)	70.4 (19.1)	71.9 (18.3)	70.0 (14.0)
Grooved Pegboard – Non-dominant (Total time)	76.4 (21.1)	78.2 (25.0)	76.9 (16.1)
HVLT-R Immediate Recall (Total Trials 1–3)	23.2 (4.5)	22.4 (4.5)	22.5 (4.7)
HVLT-R Delayed Recall	8.0 (2.1)	7.4 (2.6)	7.2 (2.5)

**Table 2:** Single-predictor ROC analyses for 11 candidate TabCAT measures and reference HAND classification in a development sample of PWH

Predictor	Domain	n	AUC (95% CI)	Sensitivity (95% CI)	Specificity (95% CI)	Threshold
Birdwatch Total	Associative learning and memory	123	0.693 (0.576, 0.810)	61.1% (43.5, 76.9)	77.0% (66.8, 85.4)	−0.691
Match Total	Processing speed, attention and working memory	142	0.727 (0.636, 0.817)	86.0% (72.1, 94.7)	49.5% (39.3, 59.7)	−0.103
Rapid Naming Total	Speeded confrontation naming	142	0.731 (0.632, 0.830)	55.8% (39.9, 70.9)	85.9% (77.4, 92.0)	−0.694
Set Shifting Total	Executive task switching	134	0.621 (0.509, 0.732)	48.6% (31.9, 65.6)	77.3% (67.7, 85.2)	−0.498
Dot Counting Total	Verbal working memory	142	0.663 (0.564, 0.762)	72.1% (56.3, 84.7)	63.6% (53.4, 73.1)	−0.565
Favorites All Trials Total	Multimodal associative memory	142	0.659 (0.563, 0.755)	58.1% (42.1, 73.0)	67.7% (57.5, 76.7)	−0.677
Flanker Total	Cognitive control and inhibition	141	0.645 (0.547, 0.744)	55.8% (39.9, 70.9)	69.4% (59.3, 78.3)	−0.44
Line Length Threshold Score	Visuospatial discrimination	142	0.531 (0.417, 0.644)	51.2% (35.5, 66.7)	67.7% (57.5, 76.7)	−0.036
Line Orientation Threshold Score	Visuospatial discrimination	142	0.602 (0.496, 0.708)	62.8% (46.7, 77.0)	57.6% (47.2, 67.5)	−0.114
Quick Tap Total Taps	Fine motor speed and dexterity	140	0.554 (0.448, 0.660)	20.9% (10.0, 36.0)	93.8% (87.0, 97.7)	0.865
Tempo Integer Precision	Rhythm keeping	140	0.610 (0.512, 0.707)	79.1% (64.0, 90.0)	42.3% (32.3, 52.7)	0.281

Predictors are demographically adjusted z-scores of each test’s primary measure. Each row represents a separate analysis using available cases. The first four rows are the selected battery; other tests follow in source order. AUC confidence intervals use DeLong’s method; sensitivity and specificity confidence intervals use the exact Clopper-Pearson method. Birdwatch was introduced after the first 39 enrollments, contributing to unavailable scores. The reported threshold for individual tests represents the z-score cutoff for determining impairment (flag as impaired if z-score ≤ threshold).

**Table 3:** ROC performance of the four-test battery and individual selected measures against reference HAND classification in validation PWH

Predictor	n	AUC (95% CI)	Sensitivity (95% CI)	Specificity (95% CI)	Threshold
4-Test Model	150	0.819 (0.740, 0.899)	81.6% (65.7, 92.3)	70.5% (61.2, 78.8)	0.221
Match	150	0.743 (0.650, 0.836)	55.3% (38.3, 71.4)	83.9% (75.8, 90.2)	−0.882
Set Shifting	151	0.740 (0.648, 0.832)	57.9% (40.8, 73.7)	85.0% (77.0, 91.0)	−0.611
Birdwatch	151	0.724 (0.635, 0.814)	71.1% (54.1, 84.6)	64.6% (55.0, 73.4)	−0.668
Rapid Naming	151	0.673 (0.571, 0.774)	47.4% (31.0, 64.2)	80.5% (72.0, 87.4)	−0.457

Predictors are demographically adjusted z-scores of the primary measure. The four-test model uses complete cases; individual measures use their available cases. AUC confidence intervals use DeLong’s method; sensitivity and specificity confidence intervals use the exact Clopper-Pearson method. In the 4-test model, 38/150 participants meet the reference criteria for HAND. The reported threshold for individual tests represents the z-score cutoff for determining impairment (flag as impaired if z-score ≤ threshold), and the threshold for the 4-test model represents the predicted probability cutoff (flag as impaired if predicted probability ≥ threshold).

## Data Availability

Data and materials can be made available by request to the corresponding author.
